# Paleoceanography of the Late Cretaceous northwestern Tethys Ocean: Seasonal upwelling or steady thermocline?

**DOI:** 10.1371/journal.pone.0238040

**Published:** 2020-08-27

**Authors:** Eric Otto Walliser, Bernd R. Schöne

**Affiliations:** Institute of Geosciences, University of Mainz, Mainz, Germany; University of Toronto, CANADA

## Abstract

In this study we attempted to assess whether seasonal upwelling or a steady thermocline persisted at the western margin of the Tethys Ocean during the late Turonian–early Coniacian interval. For this scope, we employed novel and published stable oxygen isotope (δ^18^O) data of various organisms (bivalves, bivalves, brachiopods, fish and belemnites). New seasonally resolved temperature estimates were based on the δ^18^O record of sequentially sampled inoceramid (*Inoceramus* sp.) and rudist (*Hippurites resectus*) shells from the Scaglia Rossa and Gosau deposits of northern Italy and western Austria, respectively. Diagenetic screening was performed using reflected light, cathodoluminescence (CL), scanning electron microscopy (SEM) and stable isotope analysis. Originally preserved δ^13^C and δ^18^O values were used to characterize the lifestyle of the bivalves and detect vital effects that could have biased oxygen isotope-based temperature reconstructions. Inoceramid δ^18^O values provide–for the first time–information on temperatures of Tethyan benthic waters, which were, on average, 14.4 ± 0.6 °C and fluctuated seasonally within a range of less than 2 °C. Such a thermal regime is in line with the temperatures postulated for late Turonian boreal water masses and support the existence of a cold water supply from the North Atlantic to the Tethyan bottom. Bottom cooling, however, did not affect the shallow water environment. In fact, the rudist-based temperature estimates for shallow water environment revealed a mean annual range of 11 °C, between 24 and 35 °C (assuming a seasonally constant δ18O_w_ = 1.0 ‰), which are among the warmest temperatures recorded over the entire Late Cretaceous. Our findings, thus, suggest a strong thermal and food web decoupling between the two environments. The absence of a seasonal vertical homogenization of different water bodies suggests the existence of a steady thermocline and, therefore, contrasts with the presence of an active coastal upwelling in the region as hypothesized by previous authors.

## Introduction

Following the Aptian-early Turonian ‘supergreenhouse’ event (90–95 Ma) [1,2], the Late Cretaceous world experienced a time interval of significant global cooling [3–5]. Declining atmospheric *p*CO_2_ levels [6] and plate tectonics [4] triggered profound paleoceanographic changes in the North Atlantic sector [4,7]. Numerical climate simulations indicate that deep water production in the North Atlantic was possible during most of the Late Cretaceous [8–10], and geochemical proxy data suggest that migration of cold, high latitude water masses into the equatorial Atlantic Ocean and Central Europe occurred as early as the middle and late Turonian [11–13]. During this interval, North Atlantic waters also flooded the northwestern Tethys Basin [14] increasing the dissolved oxygen levels at the bottom of the ocean [15–17], which drove to the formation of the reddish-pinkish shale, marls and limestones of the Scaglia Rossa Formation [18–20]. A coeval increase of the δ^18^O values of western Tethyan fish teeth was interpreted as the North Atlantic cooling also affecting the upper ocean waters of the northwestern Tethys [21]. In addition, the existence of an active upwelling system in the region was hypothesized [14]. However, the δ^18^O values of coeval rudist shells from Austria suggest water temperatures of up to 35 °C in shallow water environments, i.e., among the warmest temperatures recorded during the entire Late Cretaceous [22]. Therefore, it is possible that oceanic cooling did not pervade the shallowest habitats at the northwestern margin of the Tethys and thermal decoupling (instead of upwelling) existed between the bottom and the shallow water environments during the late Turonian–early Coniacian.

A fundamental physical parameter employed by modern oceanographers to test such a hypothesis would be the vertical profiling of the temperatures during different seasons [23–25]. Coastal upwelling is mainly governed by wind stress curls and, therefore, it possesses strong seasonal character [26,27]. Hence, the vertical migration of cold bottom waters to the surface usually results in a certain degree of thermal homogenization in the water column for at least part of the year [25]. For ancient water bodies, such seasonally resolved information can be inferred from the geochemical proxy record of fossil bivalves, which coexisted (in geological terms) at different water depths. Late Cretaceous shallow water habitats were dominated by rudist bivalves, which majorly contributed to the formation of peri-Tethyan deposits [28]. Rudist shells, especially those of the elevator morphotypes [29], have been extensively used in sclerochronological studies [22,30–33]. To estimate deeper sub-annual water temperatures, inoceramid shells represent a promising proxy archive. This bivalve taxon colonized nearly all benthic Late Cretaceous marine habitats and were widely distributed in the bottom environments of the Tethys [34].

Rudists and, to a lesser extent, inoceramids have been in the focus of many paleoclimate studies. However, because these taxa lack modern representatives, the validity of their stable isotope values for paleotemperature reconstructions has been questioned in the past [32], in particular for the inoceramids [35–43]. Rudist shells often display a positive correlation between δ^13^C and δ^18^O values. In a previous study, this correlation was interpreted as a sign for kinetic isotope effects, which prompted the authors to assume disequilibrium fractionation for the rudist species *Torreites sanchezii* [32]. More recent structure morphological and sclerochronological evidences, however, strongly indicate that such stable isotopic patterns derived from a photosynthetic lifestyle [33,44]. It is known that symbiotic algal activity can strongly affect the δ^13^C signal of shell [45]. However, photosynthesis does not affect the equilibrium incorporation of oxygen isotopes [45], and photosymbiotic bivalves (e.g., tridacnids) are considered among the most faithful recorders of tropical surface water temperatures (SST) [46–48]. A photosynthetic lifestyle has also been proposed for inoceramids, because some species show morphological traits comparable to modern photosynthetic bivalves [49] and might have lived pseudo-planktonic anchored to floating objects [50]. Yet, the broad environmental tolerance and preferential occurrence of this taxon in rather deep habitats make this assumption not applicable to all inoceramid species [51]. Equilibrium fractionation of oxygen isotopes between shell and water has been demonstrated for several inoceramid taxa [52–55]. However, previous studies have also hypothesized disequilibrium fractionation related to species-specific vital effects [36,43]. This is because inoceramids often display higher δ^13^C values and (sometimes) lower δ^18^O values than those of coeval organisms [35–43]. Such isotopic signatures are observed in those inoceramids that colonized dysoxic and H_2_S-rich environments (stagnant bottom waters or cold seeps) [43,52,54,56,57], and mimic the δ^13^C and δ^18^O signals of modern thioautotrophic bivalves [58–61]. In the light of the above, an assessment of the metabolisms of the studied rudist and inoceramid bivalve is required to exclude possible vital effects on the δ^18^O records, which can, ultimately, bias the estimation of the paleowater temperatures.

**Fig 1:**
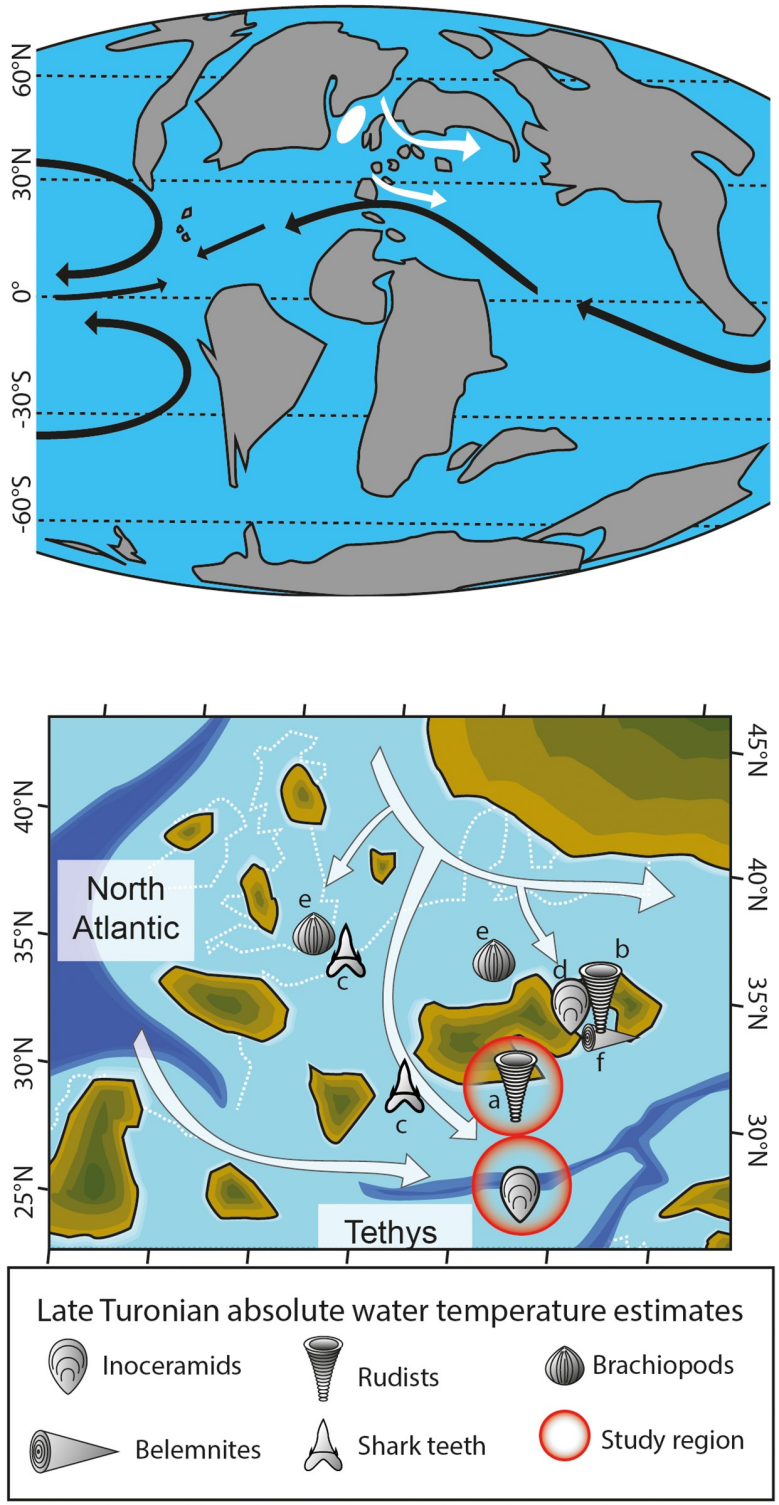
Simplified paleogeography of Earth and the northwestern margin of the Tethys during the late Turonian-early Coniacian interval. (A) Global paleogeography during the Late Turonian (modified from [62]) showing the patterns of the main surface oceanic currents (black arrows). The location of the deep water formation site in the North Atlantic (white circle) [14] and inferred ingression paths of high latitude cold waters onto the Central European shelves and the northwestern Tethys (white arrows) are shown. (B) Paleogeography of the northwestern Tethys showing possible paths of boreal and North Atlantic water ingression in the study region. The location of the ‘Atzl Reef’ and the Trento Plateau (i.e., the studied environments) are indicated by the red circles. Symbols show the location of published water temperature estimates in the region derived from the stable oxygen isotope values of (a-b) rudist shells and shark teeth as well as from (d) inoceramid, (e) brachiopod and (f) belemnite remains (cf. Table 2).

In the present study, we sclerochronologically sampled δ^13^C and δ^18^O values of well-preserved rudist and inoceramid shells to estimate the sub-annual temperature variability that prevailed in the shallow and deeper water environments of the northwestern Tethyan Ocean during the Late Turonian-Early Coniacian interval (Fig 1). Shallow water seasonal variability was reconstructed by combining novel and published sclerochronological stable isotope data of rudist material from shallow water deposits of the Gosau Formation of Austria. We also present the first temperature reconstruction of the seafloor water temperature that prevailed in the hemipelagic environment of the Tethys. Seasonal temperatures were estimated from sequentially sampled δ^18^O values of *Inoceramus* sp. shells from the ‘lastame’ deposits of the Scaglia Rossa Formation of northern Italy. Prior to using the shells for environmental reconstructions, we performed a detailed diagenetic screening of the shell material by employing a combination of cathodoluminescence (CL), scanning electron microscopy (SEM) and stable isotope analysis of different carbonate phases. Well-preserved δ^13^C and δ^18^O values were used to discuss the autoecology of the bivalves and the impact of possible vital effects on the paleowater temperature estimates. Additionally, we combined our results with previously published temperature reconstructions of coeval seawaters of the Central European basins to gain information on the vertical temperature gradient of the studied water body. Finally, temperature estimates were used to test the hypothesis of a thermal decoupling between hemipelagic and shallow water environments in study region. The present study highlights the potential of bivalve sclerochronology for palaeoceanographic studies.

### Geological settings, material and methods

**Fig 2:**
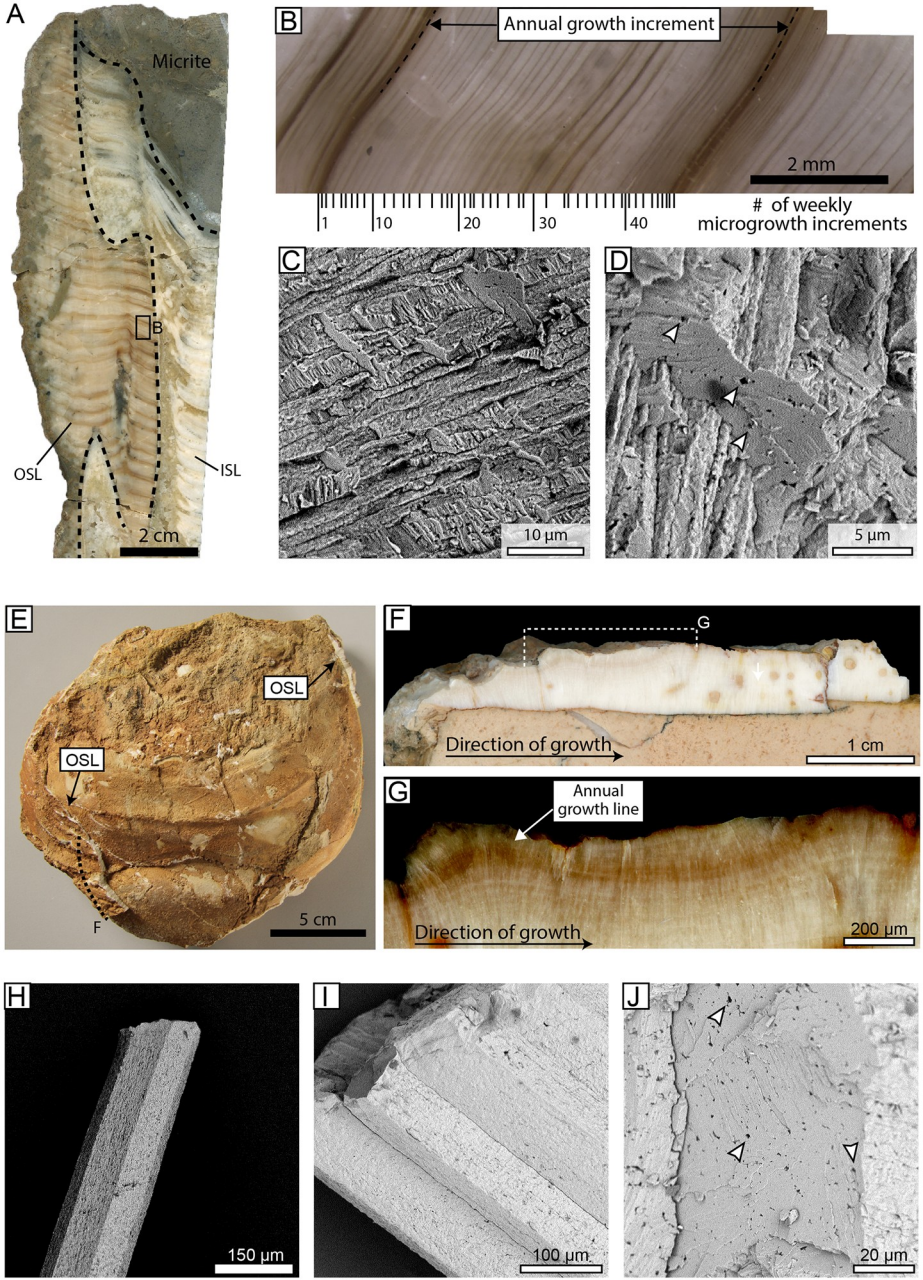
The studied shell material. (A) Cross-section trough the *Hippurites resctus* shell from the ‘Atzl Reef’ deposits of Brandenberg (Austria) showing marked annual growth lines in the outer shell layer (OSL). (B) At higher magnification, microgrowth increments were easily recognizable. Their number within one annual growth increment (ca. 49) suggests weekly shell growth cycles for the studied *H*. *resectus* individual. (C) SEM imaging revealed the preservation of the original fibrous prismatic ultrastructure of the OSL and the presence of intra-prismatic voids inside the biomineral units. (E) One of the studied *Inoceramus* sp. fossils from the ‘lastame’ deposits of Sant´Anna d´Alfaedo (Italy). The inoceramid steinkern is partially covered by original OSL. The dotted line indicates the location of the shell cross-cut depicted in (F). In cross-cut very faint annual growth lines were recognized (F), however, these became more visible after increasing the contrast of the images (G). Under the SEM, the low-Mg calcite prisms of the studied inoceramids displayed smooth surfaces and sharp edges (H) and the absence of neomorphic precipitates between adjacent prisms. (J) The inoceramid prisms also displayed intra-prismatic voids, which were originally filled with organic matrix. ISL = inner shell layer.

In the present study we sclerochronologically analyzed the shells of two *Inoceramus* sp. (ID: SNSB-BSPG 1973 VII ?60 A, B) and one *Hippurites resectus* (ID: SNSBBSPG1991X) specimens (Fig 2) stored at the Bavarian State Collection for Paleontology and Geology of Munich. Permission for all analysis performed in this study was granted by the curator of the collection and specimens were permanently stored (under the above mentioned ID-numbers) and made publicly accessible at the Bavarian State Collection for Paleontology and Geology of Munich (Richard-Wagner-Str. 10, 80333 Munich, Germany). The rudist shell was 15 cm long and exhibited distinct growth lines in its outer shell layer (OSL) (Fig 2A). The shell originates from the mixed siliciclastic-carbonate deposits of the ‘Atzl Reef’ (Gosau Group) exposed near the town of Brandenberg (North Calcareous Alps, Austria). According to paleontological and petrological studies, the limestones of the ‘Atzl Reef’ were deposited in a shallow subtidal environment [63,64], and previous δ^18^O-based temperature reconstructions suggest very warm conditions compatible with a Late Cretaceous shallow water habitat [22]. To estimate the full seasonal temperature amplitude that prevailed in this environment, the stable isotope data of our specimen were combined with those of previously published *Vaccinites inaequicostatus* shells [22,65] from the ‘Atzl reef’ and the strata exposed near the town of Theresienstein (Salzburg, Austria).

The inoceramid specimens consisted of two internal molds partially covered by fragments of the original OSL (Fig 2E). The specimens were collected from the deposits of the Scaglia Rossa Formation near the town of Sant´Anna d´Alfaedo (Lessini Mountains, northern Italy). The pinkish nodular limestone surrounding the inoceramid allows to constrain the stratigraphic position to the ‘lastame’ lithozone [66–70]. The studied succession was deposited from the late Turonian to the Coniacian on the Trento Plateau, a hemipelagic rise located at the northern margin of the Tethys few hundreds of km south of the ‘Atzl Reef’ [66]. Existing estimates of the paleobathymetric range have yielded contrasting results. The occurrence of globotruncanids and the absence of benthic foraminifera in the sediment have been interpreted as suggesting bathyal depths well below the neritic zone [71]. Some authors postulated that the Trento Plateau was situated above the local oxygen minimum zone and suggested water depths between 400 and 700 m [72]. In a more recent study, the presence of radiolitid rudists in the ‘lastame’ deposits has been interpreted as indicative for water depths near the lower photic zone (ca. 100–150 m) [67].

The studied Gosau deposits belong to the late Turonian ammonite *Subprionocyclus neptuni* and *Prionocyclus germani* zones [73] and strontium isotope values of well-preserved rudist shells from the two localities revealed an upper Turonian-lowermost Coniacian age for the ‘Atzl Reef’ and an uppermost Turonian-lower Coniacian age for the Theresienstein material [74]. The ‘lastame’ deposits span from the upper *Marginotrucana scneegansi* to the lower *Dicarinella concavata* planktonic foraminifera zones and are comprised in the nannofossil zone CC12 or UC8[69], which are temporally equivalent to the *S*. *neptuni* zone [75]. Accordingly, the studied time interval can be constrained to about 2 Ma, between 89 and 91 Ma [75].

### Shell preparation, sampling and stable isotope analysis

Fragments were first coated with WIKO metal epoxy resin to avoid breakage during cross-sectioning. Then, a 1 cm-thick slab was cut from the specimen along the direction of growth (Fig 2A, 2B, 2E and 2F). The slabs were finally ground with SiC powders (F300, F800, F1200 grit) on glass plates and polished with a 1 μm Al_2_O_3_ powder on a Buehler G-cloth.

The state of preservation of the material was evaluated visually, by means of reflected light microscopy as well as using cathodoluminescence (CL) and scanning electron microscopy (SEM). CL microscopy is a non-invasive technique, which uses a high-energy electron beam to identify the distribution of trace elements in minerals [76,77]. For example, low-Mg calcite (LMC) shells with high concentrations of Mn emit a bright orange to reddish CL and are typically regarded as diagenetically altered [53]. SEM analysis was performed on hand-broken fragments of the OSLs to evaluate the preservation of the shell ultrastructures and possible signs μm-scale recrystallization and precipitation of neomorphic material [36,78]. Finally, the stable isotope values of different biotic and abiotic carbonate phases were used to evaluate the effect of diagenesis on the original δ^13^C and δ^18^O record of the studied bivalve shells.

Carbonate powders (50–120 μm) for δ^13^C and δ^18^O measurements were collected from the biogenic LMC, from the micrite, and from sparite encrusting the shells and infilling voids (S1 Fig) using a Rexim Minimo dental drill equipped with a 300 μm-diameter conical SiC bit (Gebr. Brasseler GmbH & Co. KG, model number H52 104 003) mounted on a stereomicroscope. To ensure an adequate temporal resolution for seasonal environmental reconstructions, the inoceramid LMC was sampled from the outer to inner surface of the fragment [54], whereas the rudist OSL was sampled following the direction of growth.

Stable carbon and oxygen isotope measurements were performed at the Institute of Geoscience of the University of Mainz. Carbonate powder were digested with a water-free phosphoric acid in He-flushed exetainers at 72 °C. The liberated CO_2_ gas was then measured in continuous flow mode with a Thermo Finnegan MAT 253 gas source isotope ratio mass spectrometer coupled to a Gas Bench II. Data calibration was performed against a NBS-19 calibrated Carrara Marble distributed by IVA Analysensysteme GmbH (δ^13^C = +2.01 ‰; δ^18^O = -1.91 ‰). Stable isotope values are given in the δ-notation in parts per mil (‰). The 1σ accuracy (based on long-term analysis) and average 1σ internal precision (based on 8 injections per analyzed sample) for δ^13^C values were better than 0.04 and 0.02 ‰, respectively, and better than 0.05 ‰ and 0.03%, respectively, for δ^18^O values.

### Estimation of water temperatures and δ^18^O signal

Since bivalve shells are formed near isotopic equilibrium with the ambient water, their δ^18^O values can be used to reconstruct paleowater temperatures [79]. The OSL of rudists and inoceramids was composed of fibrous and prismatic LMC, respectively. Both taxa also produced an aragonitic inner shell layer, which was not preserved in studied specimens. Therefore, in the present study, we employed a paleothermometry equation [80], which is typically used for rudist and inoceramid LMC [22, 45]:

$$T_{\delta 18O}({}^{\circ}C)=16.0-4.14\times (\delta^{18}O_{shell}-\delta^{18}O_{w})+0.13\times {\left(\delta^{18}O_{shell}-\delta^{18}O_{w}\right)}^{2}$$ (1)

whereby, the δ^18^O_shell_ and δ^18^O_w_ values are given relative to the VPDB and VSMOW scale, respectively. According to Eq (1), the computation of δ^18^O-based paleotemperatures requires the knowledge of the water δ^18^O signature (δ^18^O_w_). In the present study, we performed temperature calculations considering different δ^18^O_w_ scenarios. For the Late Cretaceous ice-free world, an average oceanic δ^18^O_w_ signal of -1.0 ‰ is typically assumed [81], whereas, climate model simulations suggest an average value of +0.5 ‰ for the Cretaceous Tethyan surface waters [82]. Furthermore, we constructed a model to assess the possible effects of the δ^18^O_w_ changes in the water column on the reconstruction of the vertical temperature gradient. We simulated four scenarios by fitting polynomial functions to observational δ^18^O_w_ data from the first 900 m water depths of tropical Indian, Pacific and Atlantic Ocean as well as the Gulf of Mexico [83]. Additionally, we employed the Late Cretaceous vertical δ^18^O_w_ gradient simulated using the GENESIS-MOM numerical climate model [82]. The modeled gradients were used to calculate the vertical temperature amplitude (ΔT) required to justify the shell δ^18^O_shell_ difference between the surface (10 m) and bottom environments (900 m water depth).

## Results

### Rudist material

**Fig 3:**
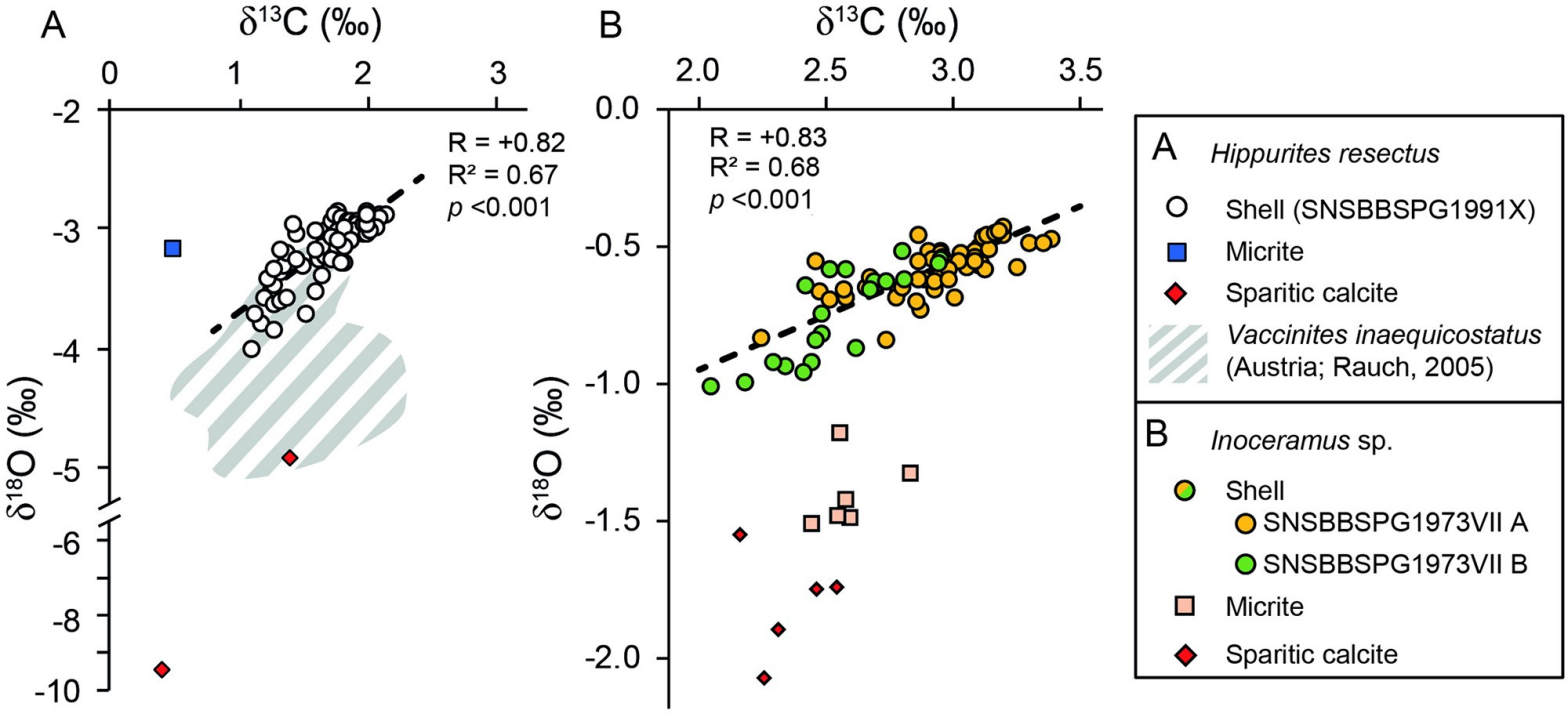
Plots of the δ^13^C and δ^18^O values of the carbonates analyzed in this study. These include burial sparite, micrite, and the low-Mg calcite shells of the studied inoceramid and rudist material. (A) The stable isotope values of the *Hippurites resectus* shell are positively correlated (R = +0.82; R^2^ = 0.67; *p*-value < 0.001) and plot in the upper range of previously published δ^13^C and δ^18^O values of coeval the *Vaccinites inaequicostatus* rudists [65] (shaded area). (B) The studied *Inoceramus* sp. specimens also display a positive correlation (R = +0.83; R^2^ = 0.68; *p*-value < 0.001) and does not overlap with the stable isotope values of diagenetic altered phases (micrite and sparite).

**Fig 4:**
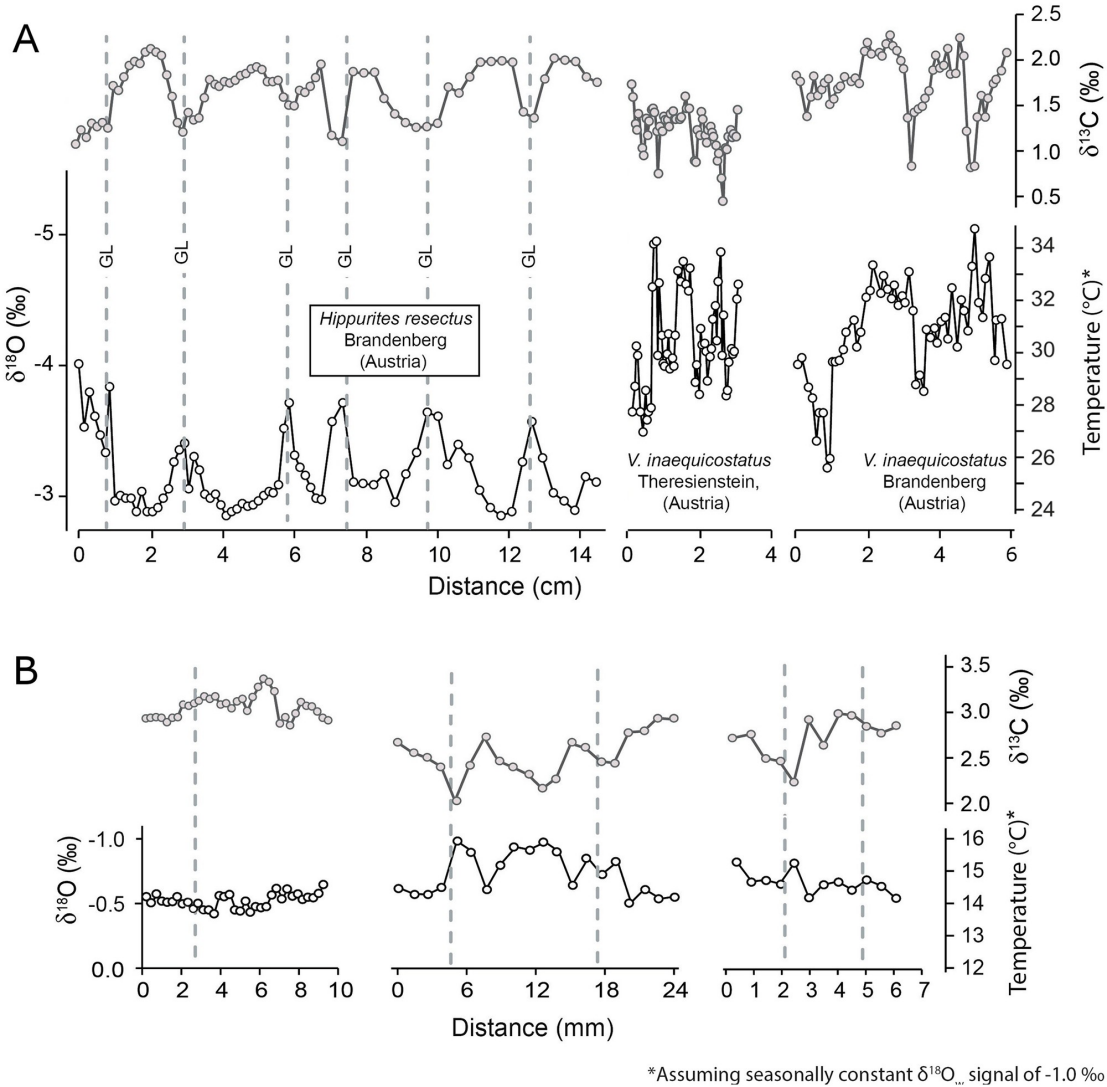
Sequentially sampled δ^13^C and δ^18^O values the studied rudist and inoceramid shells and temperatures reconstructed thereof. (A) In the *Hippurites resectus*, the position of the growth lines (GL; gray dotted lines) coincided with the δ^18^O minima, which suggest that shell growth occurred mostly during winter. In comparison, the shape of the δ^18^O curves of the coeval *Vaccinites inaequicostatus* [65] suggest a predominant summer shell growth. (B) In the *Inoceramus* sp. specimens, growth lines were located shortly before the seasonal δ^18^O minima, suggesting that the full seasonal temperature amplitude was recorded by the bivalves. Temperatures were computed assuming an average ocean δ^18^O_water_ value of -1.00 ‰ [81].

The OSL of the *Hippurites resectus* specimen exhibited marked dark brown major growth lines, which divided the shell growth pattern into 4–5 mm broad growth increments (Fig 2A). In turn, major growth increments were subdivided by thin microgrowth lines into 40–50 microgrowth increments of 20–100 μm width (Fig 2B). Under the CL, the OSL was non-luminescent, and SEM imaging revealed the preservation of the original fibrous calcitic ultrastructure of the shell. Occasionally, μm-scale neomorphic precipitates were observed on individual calcitic fibers. The presence of intra-crystalline voids, however, suggest that recrystallization remained only very superficial (Fig 2C and 2D). Orange to bright red luminescence was emitted by the sparitic cements, which replaced the original aragonitic shell layer of the rudist and exhibited the most negative δ^18^O values (-9.38 ‰) (Table 1). The δ^18^O value of the micrite (-3.18 ‰) fell within the range of stable isotope oxygen values measured in the OSL (-4.92 to -2.85 ‰). The micrite δ^13^C value (0.46 ‰) was slightly lower than those measured in the biogenic LMC (δ^13^C = 1.08–2.12 ‰) (Fig 3A; Table 1). Sequentially sampled δ^13^C and δ^18^O data from the OSL were positively correlated (R = +0.82; R^2^ = 0.68; *p* >0.01) (Fig 3A) and exhibited five consecutive oscillations, with the major growth lines coinciding with the δ^18^O and the δ^13^C minima (Fig 4A).

**Table 1:** Stable oxygen and carbon isotope values of the different carbonate phases analyzed in this study.

			δ^13^C_shell_			δ^18^O_shell_		
Bivalve taxon (specimen ID)	Carbonate phase	Min (‰)	Average ± 1 σ (‰)	Max (‰)	Min (‰)	Average ± 1 σ (‰)	Max (‰)	# of isotope samples
*Inoceramus* sp. SNSB-BSPG 1973 VII ?60	Sediment	2.44	2.60 ± 0.12	2.83	-1.50	-1.32 ± 0.23	-0.86	6
	Sparitic calcite	2.17	2.35 ± 0.15	2.55	-2.07	-1.80 ± 0.19	-1.55	5
	Shell A							
	OSL Fragment 1	2.86	3.07 ± 0.13	3.38	-0.65	-0.52 ± 0.05	-0.42	35
	OSL Fragment 2	2.24	2.73 ± 0.19	3.00	-1.44	0.71 ± 0.22	-0.45	24
	Shell B	2.05	2.54 ± 0.24	2.95	-1.00	-0.74 ± 0.17	-0.51	21
*Hippurites resectus* (SNSBBSPG1991X)	Sediment	-	0.46	-	-	-3.18	-	1
	Sparitic calcite	-0.39	-0.88	1.39	-9.40	-7.16	-4.92	2
	OSL	1.08	1.67 ± 0.28	2.12	-4.02	-3.18 ± 0.28	-2.85	73

Stable isotope values are given for the sediment, the sparitic calcite and the shells of the *Inoceramus* sp. and *Hippurites resectus* specimens.

### Inoceramid material

The inoceramid shells exhibited sharply defined polyhedral prisms, and faint major growth lines were identified when viewed under a reflected-light microscope (Fig 2F and 2G). Under CL, the studied inoceramid shell fragment was non-luminescent to dark blue luminescent, whereas, orange luminescence was emitted by the micrite and the sparite infilling the foraminiferan tests. The latter exhibited the lowest δ^13^C (+2.35 ± 0.14 ‰; ± 1σ) and δ^18^O values (-1.80 ± 0.17 ‰; ± 1σ), whereas slightly more positive values were measured in the micrite (δ^13^C = +2.60 ± 0.11 ‰; δ^18^O = -1.32 ± 0.22 ‰; ±1σ) (Fig 3B; Table 1). Under the SEM, LMC prisms were tightly packed, and no neomorphic precipitates were observed within the inter-prismatic space (Fig 2I). The individual biomineral units displayed smooth surfaces and sharp edges, and preserved intra-crystalline voids, visible in cross-sections (Fig 2H and 2J). The inoceramid LMC yielded the most positive δ^13^C and δ^18^O values, which were, on average, +2.83 ± 0.13 ‰ and -0.61 ± 0.13 ‰ (± 1σ), respectively (Fig 3B; Table 1). Shell δ^13^C values fluctuated between +2.04 and +3.38 ‰, whereas the δ^18^O data oscillated within a much narrower range, i.e., between -0.99 and -0.42 ‰ (Fig 4B). The δ^13^C and δ^18^O data were positively correlated (R = +0.83; R^2^ = 0.68; p < 0.01) (Fig 3B), and the faint growth lines were located shortly after the local maxima (Fig 4B).

### Water temperature estimates

In combination with previously published δ^18^O_shell_ values of the rudist *Vaccinites inaequicostatus* from the same locality and slightly younger Gosau deposits [22,65], our *H*. *resectus* data suggest an environmental δ^18^O amplitude of 2.28 ‰ (-5.10 to 2.85 ‰). Assuming a seasonally constant δ^18^O_w_ of -1.00 ‰, these δ^18^O values translate into SST between 24 and 35°C (Fig 4A; Table 2). Temperature estimates become 7–8 °C higher (31 to 43 °C) if a δ^18^O_w_ value of +0.5 ‰ [82] is applied. The δ^18^O values of the studied inoceramid shell yielded paleowater temperatures between 13–14 and 16 °C (Fig 4B) as well as 21 and 23 °C assuming a δ^18^O_w_ value of -1.0 ‰ and +0.5 ‰, respectively. Hence, our temperature estimates suggest an average vertical ΔT value of 14–15 °C between the shallow and bottom water environment if a constant vertical δ^18^O_w_ value is assumed. Simulated vertical changes in δ^18^O_w_ signals suggest vertical ΔT gradients between 14 and 19 °C, whereby the assumed water depth plays an important role in the Atlantic and Gulf of Mexico scenario. The Late Cretaceous and Indian Ocean settings yielded the most conservative ΔT outputs (13–14 °C), which are close to the ΔT value calculated assuming a constant δ^18^O_w_ value.

**Table 2:** Temperature estimates used for the reconstruction of the vertical water temperature gradient at the northwestern margin of the Tethys.

Environment (water depth)	Proxy archive (Taxon)	Locality	Age	Reference	T_δ18O_		
					Min (°C)	Average ± 1σ (°C)	Max (°C)
**Shallow water (2–10 m)**	Rudist OSL (*Hippurites resectus*)	‚Atzl Reef‘, Brandenberg (Austria)	Late Turonian/ earliest Coniacian	**This study**	24.1	25.6 ± 1.3	29.7
	Rudist OSL (*Vaccinites Inaequicostatu*)	‚Atzl Reef‘, Brandenberg (Austria)	Late Turonian/ earliest Coniacian	[22,65]	25.9	30.9 ± 1.9	35.0
	Rudist OSL (*Vaccinites Inaequicostatu*)	Theresienstein Strobel (Austria)	Latest Turonian/early Coniacian	[22,65]	27.2	30.6 ± 1.8	34.4
	Rudist OSL (*Radiolites undulatus*)	Kučlín (Czech Republic)	Turonian	[84]	32.9	33.8	34.7
**Upper ocean water (<150 m)**	Fish tooth enamel and enameloid (Lamniforme, undetermined)	Somme, Adèrche (France)	Turonian, Coniacian	[21]	21.0	24.0	28.4
**Central European shelf (50-150m)**	Inoceramid OSL (*Inoceramus hercules*)	Úpohlavy warking quarry (Czech Republic)	Late Turonian	[55]	16.2	19.7 ± 1.0	24.9
	Brachiopod shell* (rhynchonellids, terebratulids)	Dover, Kensworth, Lewes (southern England); Söhlde, Hoppenstedt (northern Germany).	Late Turonian	[12]	16.0	18.6 ± 1.1	22.0
	Belemnite rostrum (*Praeactinocamax bohemicus*)	Úpohlavy warking quarry (Czech Republic)	Late Turonian	[85]	16.4	14.8	15.2
**Hemipelagic water (Trento Plateau) (150–200 m)**	Inoceramid OSL (*Inoceramus* sp.)	Sant´Anna d´Alfaedo (Italy)	Late Tur./ Con.	**This study**	13.6	14.4 ± 0.6	16.0

Water temperatures were compiled using novel and published skeletal δ^18^O data. Temperatures were estimated assuming an average water δ^18^O signature of -1.00 ‰ [81]. Brachiopod-derived temperature should be considered with caution, because these organisms may have not fractionated oxygen isotopes in equilibrium with the ambient water.

## Discussion

### State of preservation

The reconstruction of paleowater temperatures from δ^18^O values requires a detailed diagenetic screening of the biogenic carbonates. This is because accidental sampling of diagenetically altered shell portions can lead to an overestimation of the actual paleowater temperature. The OSL of both analyzed bivalve species consisted of LMC, which is a carbonate polymorph considered to be fairly resistant against diagenetic alteration. As suggested by the presence of original macrogrowth and microgrowth lines, the studied shells did not undergo major recrystallization during burial. Likewise, at a microscopic scale we did not observe mineral modification. The SEM analysis of the rudist and the inoceramid OSL revealed well-defined fibrous prismatic (Fig 2C and 2D) and prismatic (Fig 2H–2J) aggregates, respectively. Particularly, the presence of intra-prismatic voids (Fig 2D and 2J), which are supposed to have been originally filled with intra-crystalline organic matter [86,87], suggest that alteration of the biomineral units (if present) remained only very superficial. In previous sclerochronological studies, this feature was considered to be indicative for very good preservation and a discriminant factor for δ^18^O-based temperature reconstructions [31,32,86,87].

The well-developed δ^13^C and δ^18^O cycles measured in the *Hippurites resectus* specimen (Fig 4A) allowed us to confidently exclude a resetting of the original geochemical signal during burial [88]. Hence, the stable isotope values were considered to reflect environmental seasonality. The narrow δ^13^C (1.0 ‰) and δ^18^O (0.40 ‰) fluctuations measured in the inoceramid shells required a more detailed evaluation. In fact, such reduced stable isotope amplitudes can also result from geochemical resetting. The sparite, which exhibited the lowest δ^13^C and δ^18^O values, is typically formed during burial and directly precipitates from pore fluids [89]. Therefore, its stable isotope values can be considered as the isotopic endmember of the diagenetic alteration. Accordingly, increasingly altered carbonate material should show a progressive trend toward the δ^13^C and δ^18^O values of the sparite [90]. Such trend was visible in the micrite (Fig 3B), which exhibited δ^13^C values (+2.60 ± 0.11 ‰) coherent with the bulk stable carbon isotope data of other late Turonian-Coniacian Scaglia Rossa successions from Central Italy [91]. The relative uniformity between the isotopic signal of the micrite and the sparite suggests a closed diagenetic system, in which the stable isotope composition of the pore fluids was dominated by the dissolution of the host rock [92]. Such a condition greatly increases the preservation potential of the original stable isotope signature of biogenic calcite [92]. This is also suggested by the fact that inoceramid δ^18^O and (partially) δ^13^C values were more positive than those of the micrite. Accordingly, isotopic exchange between LMC and pore fluid during burial was likely subordinated, and possible minor changes in the original stable isotope signature remained within the analytical uncertainty. Furthermore, the measured stable isotope oscillations were similar to those observed in other Late Cretaceous bivalves (including inoceramids) [43,53,93] living in deeper settings. In the light of the above, the inoceramid δ^13^C and δ^18^O values were considered to reflect the near-original signature and, therefore, suitable for paleoenvironmental reconstructions.

### Stable isotope seasonality in the studied environments

#### The shallow water environment

The striking feature of the stable isotope record of the studied *Hippurites resectus* shell is the robust correlation between the δ^13^C and δ^18^O values (Fig 3A). It is highly unlikely that this stable isotope pattern resulted from kinetic fractionation effects, because rudists precipitated their shells in isotopic equilibrium with the ambient water [32]. Correlation between δ^13^C and δ^18^O has been observed in many rudist species [33,65] and is a typical feature observed in modern photosymbiont-bearing bivalves, e.g., tridacnids [45,46]. In photosymbiotic bivalves, such a trend results from the enhanced uptake of ^12^C from the ambient water by the symbiotic algae during periods of high insolation and warm water conditions (i.e., more negative shell δ^18^O values) [45]. Although controversially debated in the past [28], recent studies provided morphological [44] and sclerochronological [32] evidence supporting a photosymbiotic lifestyle for at least some species of elevator rudists. A further hint for a photosymbiotic lifestyle for *H*. *rectus* is given by the growth increment counting. The number of microgrowth increments between the annual growth lines (~48) is almost two-fold the number of fortnight cycles (~25) expected to have occurred during a Late Cretaceous year [94]. This suggest that the studied *H*. *resectus* bivalve grew with a weekly periodicity and formed its shell almost year-round. These two features are also usually observed in modern algae-bearing tridacnids [45] and in other supposedly photoautotrophic rudists [33].

The cuspate shape of the δ^18^O chronology and the occurrence of the growth lines at the seasonal δ^18^O minima indicates that most of the shell production occurred during the cold part of the year [95]. Shell accretion rates strongly decreased with the onset of the seasonal warming and possibly shortly ceased during the summer extremes. Thus, the measured summer δ^18^O values are strongly affected by time-averaging and do not allow to properly resolve the information on the seasonal temperature maxima that prevailed in the studied environment. Late Turonian–early Coniacian summer temperature information of the Gosau environment are provided by published δ^18^O data of sequentially sampled *Vaccinites inaequicostatus* shells [22,65]. In the frame of the present study, it not possible to precisely determine the timing of shell formation of these rudists, because the position of the growth lines was not reported by the authors, and congeneric bivalves show a large inter-specific variability in the timing of annual growth line formation [30,32,95,96]. However, as shown in Fig 4A, the δ^18^O values of *V*. *inaequicostatus* follow an opposite trend as observed in the *H*. *resectus* shell indicating a predominant summer growth season. The reduced overlapping of the δ^18^O values with those of *H*. *resectus* can be interpreted as a prolonged shell growth stop during the colder part of the year. However, because of the lower sampling resolution as employed here, the reduced overlapping of the two datasets can also be explained by the larger time averaging of the δ^18^O values near the annual growth lines. Nevertheless, the data clearly indicate the existence of contrasting shell growth strategies between the two rudists, a phenomenon also known from modern bivalve cohorts [97]. Thus, by combining the δ^18^O data from the two taxa it is possible to estimate a seasonal isotopic amplitude of 2.25 ‰ and seasonal extremes of -5.10 ‰ and -2.85 ‰, for the studied shallow water setting.

#### The hemipelagic environment

The validity of inoceramid stable isotope data for paleoclimate reconstructions has been questioned in the past. This is because some taxa likely harbored symbionts, which affected their stable isotope data [43,52,54]. Because of the low or even absent light intensity excepted at the inferred water depth of the Trento Plateau, a photosynthetic lifestyle for studied *Inoceramus* sp. is unlikely. However, even if present, a photoautotrophic metabolism does not represent an obstacle to the reconstruction of paleowater temperatures, because algal activity does not affect the δ^18^O values of the bivalve hosts [47,48]. The pervasive presence of authigenic hematite [15–17] in the studied deposits clearly indicate the existence highly oxygenated waters [18–20]. Therefore, the presence of high levels of H_2_S and dysoxic conditions near the sea floor can be confidently excluded. Such conditions, however, were indispensable to sustain a thioautotrophic metabolism. Thus, studied *Inoceramus* sp. shells were likely not subject to chemosymbiotic disequilibrium fractionation effects.

**Fig 5:**
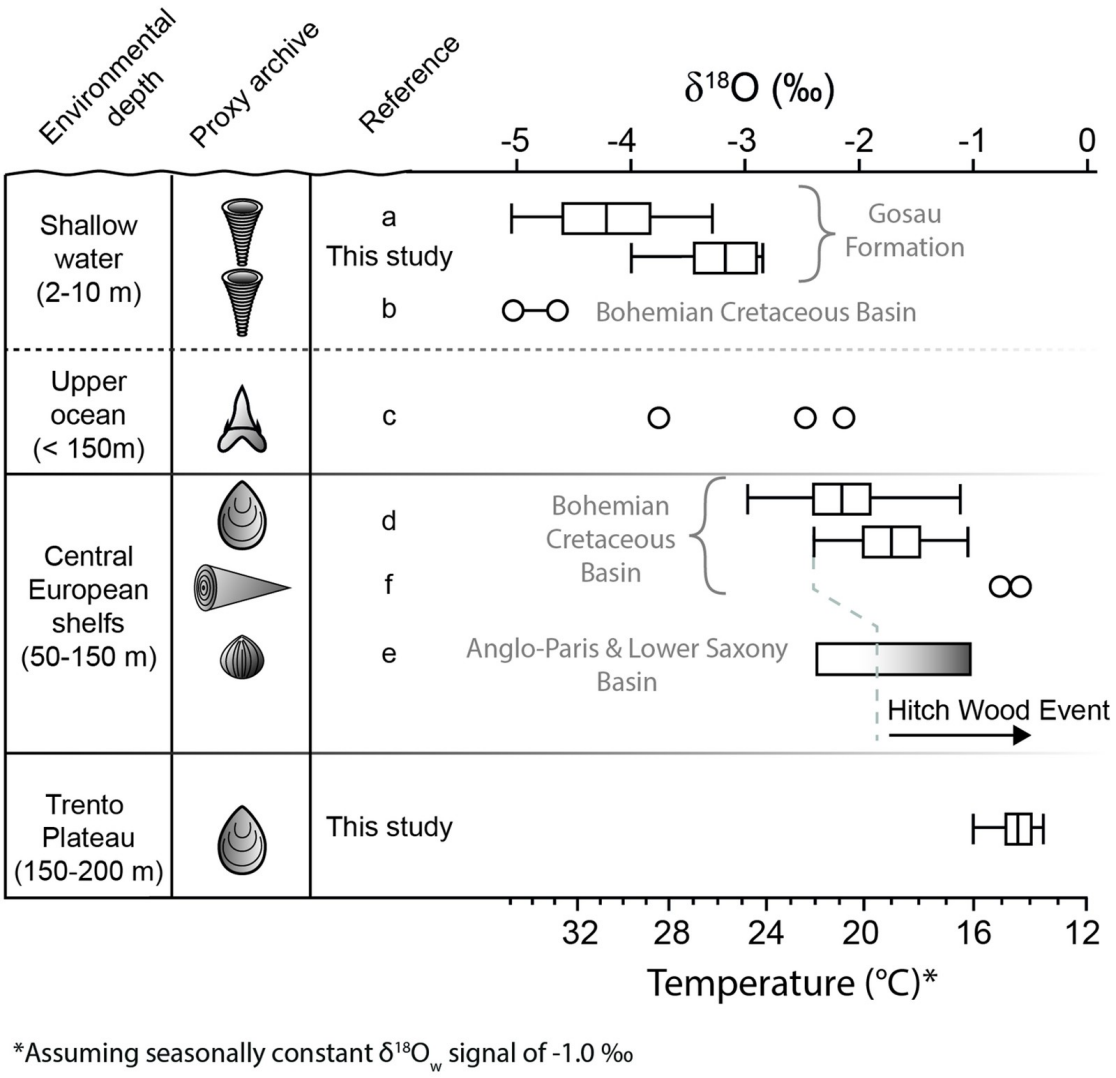
The reconstructed vertical temperature gradient at the northwestern margin of the Tethyan Ocean. Values were compiled from novel and published data. Seasonally resolved temperature estimates are shown as box plots, whereas temperature ranges are shown as bars. Dots represent the temperature estimates based on single δ^18^O values. Temperature estimates for the shallow and upper ocean waters derived from stable oxygen isotope data of (a-b) rudist shells [22,65,84] and (c) shark teeth [21]. Temperature information for deeper environments were derived from (d) inoceramid [55] and (e) brachiopod shells [12] as well as from a (f) belemnite rostrum [85]. The Hitch Wood Event represents the coldest interval of the late Turonian. Temperatures were computed assuming an average ocean δ^18^O_water_ value of -1.00 ‰ [81].

Furthermore, isotopic equilibrium fractionation is also suggested by the good agreement of δ^18^O values with those reported in the literature for other carbonate secreting organisms. The δ^18^O data of the studied inoceramid shells (-1.44 –-0.42 ‰) fall well into the δ^18^O range of late Turonian/early Coniacian North Atlantic benthic foraminifera (-1.5 –-0.6 ‰), for which equilibrium fractionation can confidently be assumed [4]. At the same time, our δ^18^O values data were slightly more positive than those measured in coeval mollusk LMC from the shelf deposits of Central Europe. Late Turonian inoceramid and belemnite skeletons from the deposits of the Hitch Wood Event (i.e., the coldest interval of the late Turonian) in the Bohemian Basin (western Czech Republic) exhibit δ^18^O values between -2.46 and -1.06 ‰ [55] as well as -0.81 and -0.62 ‰ [49], respectively (Fig 5). The belemnite data were derived from the rostrum of a *Praeactinocamax bohemicus* specimen, a belemnite species that exclusively lived within inner shelf waters of the Bohemian Basin [49]. The stable isotope signature of the *Inoceramus* sp. also compares well with the average δ^18^O values of brachiopods (-1.33 ‰) from the Hitch Wood Event deposits of the Anglo-Paris Basin and the Lower Saxony Basin [12]. A similarity between the δ^18^O values of these two organisms, however, should be taken with caution, because brachiopod δ^18^O values can be affected by non-equilibrium isotopic fractionation [12,98,99]. Our Tethyan inoceramids also display more positive δ^18^O values than those of sub-tropical oysters from younger deposits of the Western Atlantic for which isotopic equilibrium with the ambient water are very likely [100].

The measured seasonal δ^18^O fluctuations (< 0.5 ‰) are also compatible with the reduced seasonality expected for marine waters at the postulated depths (lower photic zone or deeper) of the studied environment [67,71,] and compare very well with the stable isotope fluctuations recorded by mollusks from other Late Cretaceous hemipelagic benthic environments. Seasonal δ^18^O oscillations of 0.5 ‰ were recovered from Turonian-Coniacian inoceramids from the southern North Atlantic, for which equilibrium isotopic fractionation was inferred [53]. Our δ^18^O range also compares very well with the seasonal δ^18^O fluctuations reconstructed from deep water arcoid bivalves from the Coniacian [101] and Campanian [43,93]. Proxy-development studies on modern arcoids clearly indicate equilibrium isotopic fractionation for this taxon [97,102]. Hence, in the light of the above, it can be assumed that the studied *Inoceramus* sp. bivalves formed their shells near equilibrium with the oxygen isotope signature of the ambient water, as postulated for other inoceramid species [52–55].

The analysis of the inoceramid growth patterns revealed that faint microgrowth lines were not uniformly distributed across the shell transect, but rather occurred as sporadic bundles. This suggests the absence of major seasonal variations in shell growth rate. Although not common in modern bivalves [103], year-round shell growth has also been suggested for other inoceramid species from Central Europe [55], the Western Interior Seaway of the USA [54] and the North Atlantic Ocean [53]. Furthermore, the observed growth lines were located shortly after the seasonal δ^18^O minima indicating that shell accretion rates of the studied *Inoceramus* sp. slowed down during decreasing water temperatures. Hence, the individuals captured in their shells information on both seasonal environmental extremes [95]. In other words, unlike *H*. *resectus*, the time averaging did not affect the record of the seasonal δ^18^O minima and maxima, which allows the estimation of the full annual temperature amplitude for the benthic environment of the Trento Plateau.

The fluctuation of the δ^13^C values (1.34 ‰) indicates the presence of sub-annual variations in the incorporation of carbon isotopes. Symbiotic activity is known to have a major impact on the δ^13^C of bivalve shells [45]. However, as discussed above, a symbiotic lifestyle for the studied inoceramids can be excluded. In asymbiotic bivalves, changes in δ^13^C values of bivalve shells are regarded as reflecting variations in trophic conditions and have been employed to reconstruct past primary production [104–108]. Particularly for inoceramids, δ^13^C variations were suggested to indicate changes in food availability in Cretaceous benthic environments [53,54]. This is because the particulate organic matter in seawater has a very negative δ^13^C value (< -20‰) [109,110] and contributes up to 10% of the carbon incorporated into the bivalve shells [45]. Additionally, due to organic matter remineralization, increasing concentrations of organic matter in the water column can increase ^12^C in the dissolved organic carbon (DIC) pool. The DIC, in turn, is the major source (up to 90%) of carbon for bivalve shell production [45]. Therefore, the observed δ^13^C fluctuations of the studied inoceramid shell most likely indicate seasonal fluctuations in the availability of food at the seafloor [43,45,54]. For the benthic environment of the Demerara Rise it was postulated that food supply was provided by primary producers snowing from surface waters [53].

### Water temperature estimates

Estimates of water temperature from biogenic δ^18^O data strongly depends on the assumption of a plausible δ^18^O_w_ value for the studied environment [79]. For Late Cretaceous marine waters, this assumption can be particularly challenging. Most of the existing δ^18^O-based paleothermometry studies employed an average δ^18^O_w_ value of -1.0 ‰ [22,30–33,43,53,93] assuming a Late Cretaceous ice-free world [81]. Accordingly, our shallow water δ^18^O data suggest a seasonal temperature amplitude of 11.0 °C and seasonal extremes of 24 and 35 °C (Fig 5). Such reconstructions, however, might underestimate the actual temperatures that prevailed in the studied surface environment. In fact, it should be kept in mind that the δ^18^O_w_ value of -1.0 ‰ is inferred on a global average and does not account for possible spatial heterogeneity in water isotope signatures of the Cretaceous oceans. Late Cretaceous climate simulations suggest an increase of δ^18^O_w_ values towards the equator and a δ^18^O_w_ signature ranging between +0.3 and +0.5 ‰ for the tropical Tethyan surface waters [82]. This δ^18^O_w_ signature compares well with the upper range of δ^18^O_w_ values estimated for open marine sub-tropical Gulf of Mexico (0.41–0.59 ‰) derived from carbonate clumped isotope (Δ_47_) data of Late Cretaceous mollusk shells [100]. Applying such δ^18^O_w_ values to the paleothermometry Eq (1), the estimated paleotemperatures increase by up to 6 °C. The resulting annual mean SST of 37 °C agrees with existing SST estimates from δ^18^O_w_-independent proxies. TEX_86_ data from tropical-equatorial paleolatitudes suggest annual average SSTs of ca. 35 °C during the late Turonian-Early Coniacian interval [10,111].

**Fig 6:**
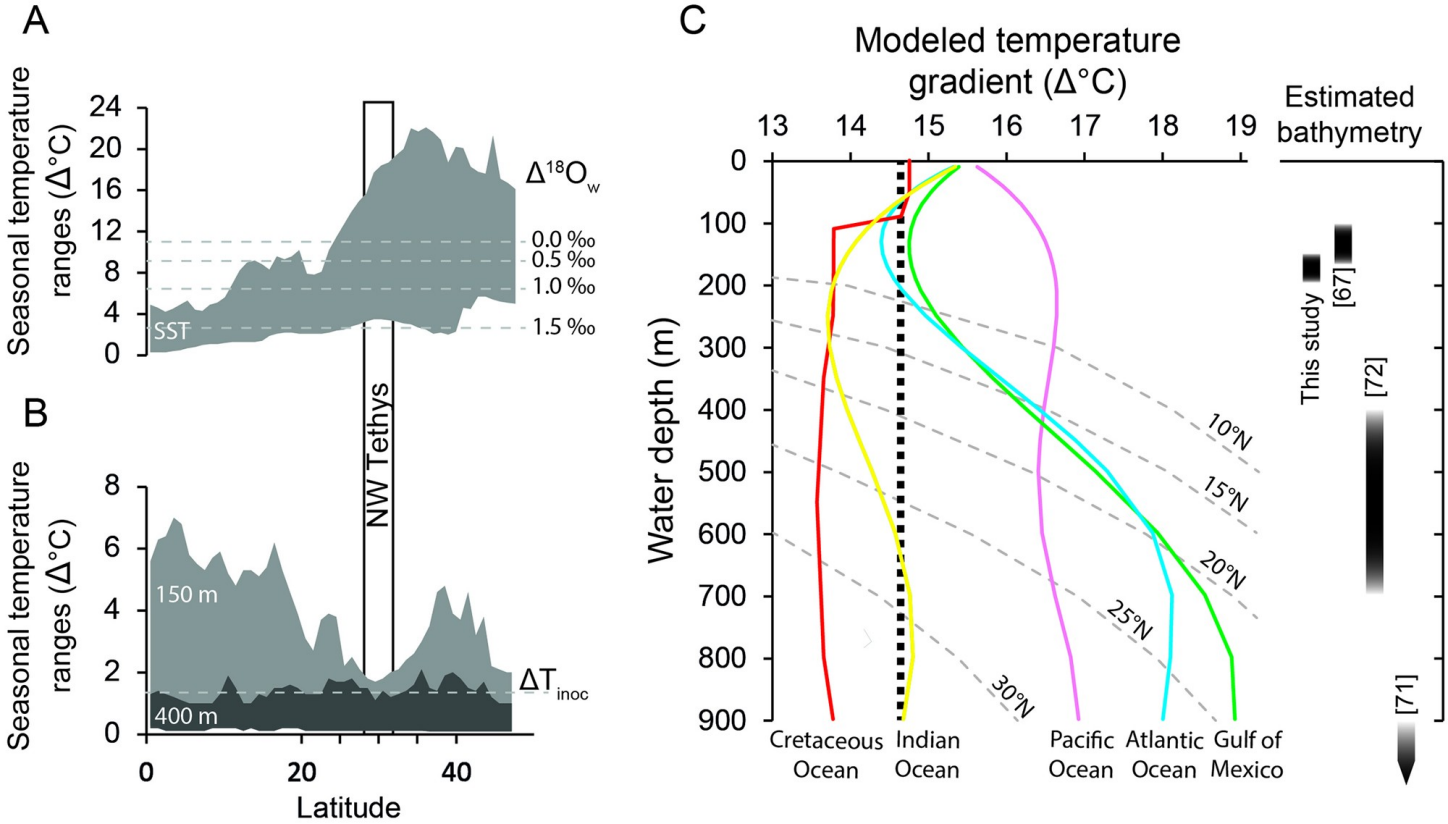
Comparison between inferred late Turonian/Early Coniacian water temperatures and observational data from modern tropical oceans. (A) Annual water temperature amplitudes (ΔT) of the studied shallow water environment of the Tethys (dashed lines) reconstructed from the sclerochronologically sampled δ^18^O values of the rudist shells (Fig 4A) are compared to the latitudinal changes of the seasonal near-surface (2.5–7.0 m) temperatures observed in modern oceans (gray shadow) [112]. When seasonal changes in δ^18^O_w_ (Δ^18^O_w_) are taken into account, the reconstructed ΔT ranges become narrower (see text for detailed explanation). (B) Similar to (A), the estimated seasonal ΔT amplitude for the hemipelagic environment was compared to observed ΔT ranges in modern oceans at 150 m (light gray) and 400 m (dark gray) water depth [112]. (C) Results of our model on the effect of bathymetrical δ^18^O_w_ changes on the reconstruction of the vertical temperature gradient (see method for detailed explanation). Vertical ΔT gradients were calculated from the rudist and inoceramid data assuming the vertical δ^18^O_w_ changes modeled for the Late Cretaceous oceans [82] as well as observed in the modern Indian (yellow), Pacific (pink), and Atlantic Oceans (blue) and the Gulf of Mexico (green) [83]. The gray dotted line depicts zonally averaged vertical ΔT gradients. Bathymetries (black bars) of the Trento Plateau inferred in the present and previous studies [67,71,72] are depicted to the right.

Seasonal SST oscillations of 11°C (assuming an invariant δ^18^O_w_ signature) have been considered to be coherent with the mid-Cretaceous greenhouse interval [22], but are larger than the maximum annual SST range observed at modern tropical latitudes [112] (Fig 6A). However, it can be presumed that late Cretaceous surface waters experienced seasonal changes in the δ^18^O_w_ signature, which should be considered in δ^18^O-based temperature estimates. Assuming a tropical monsoon climate similar as today, the cumulative effect of enhanced precipitation (lowering of the δ^18^O_w_ signal) and increasing SSTs, and *vice versa* enhanced evaporation (rising δ^18^O_w_ signal) and decreasing SSTs, would result in a dampening of the estimated annual temperature ranges (Fig 6A). For example, assuming a seasonal δ^18^O_w_ fluctuation of 1.5 ‰, as observed in some modern tropical seas [83], our carbonate δ^18^O data would yield into an annual SST amplitude of 3.5 °C (Fig 6A).

Assuming a δ^18^O_w_ signature of -1.0 ‰, the inoceramid δ^18^O data translate into temperatures ranging between 13–14 and 16 °C for benthic waters on the Trento Plateau, which fall in the lower range of temperatures estimated for the Central European shelves during the Hitch Wood Event (14–21°C) [12,55,85]. Climate simulations, however, indicate that δ^18^O_w_ could have increased by up to 0.4 ‰ in the first hundreds of meters below sea surface [82]. Assuming a similar trend for the Tethys ocean, δ^18^O_w_ values in the upper water body of the study region might have been as high as 0.9 ‰, shifting our δ^18^O-based estimates to 22–26 °C. Bivalve Δ_47_ data from younger Late Cretaceous deposits of North America suggest similar temperatures (22–23 °C) for open marine waters of the Gulf of Mexico. Therefore, it is possible that Tethyan deep waters δ^18^O_w_ were more positive than previously assumed. On a seasonal scale, temperature oscillations of < 2 °C are in line with data from other Late Cretaceous hemipelagic environments [43,53,93,101]. Furthermore, our ΔT estimates fall within the annual temperature amplitude observed in modern tropical oceans between 150 and 400 m water depth (Fig 6B), where sub-annual variations in δ^18^O_w_ are very constrained [83]. Therefore, it can be assumed that short-term variations of the water oxygen isotope signature had only a minor effect on δ^18^O-based temperature estimates.

In the context of the present study, we focused on the assessment of vertical temperature changes in the water column, and less on absolute reconstructions. However, the uncertainties in the temperature reconstructions highlighted above outline the importance to implement climate research with other proxy data.

### Vertical temperature gradient and paleoclimate implications

The reconstructed seasonal δ^18^O amplitudes suggest that rudists and inoceramids were exposed to waters with very different physical properties, e.g., different δ^18^O_w_ signatures, temperatures, or a combination of both factors. As mentioned above, vertical changes in the δ^18^O signal of the water could have played a role during the incorporation of oxygen isotopes. However, this effect was likely not dominant. In fact, to fully justify the 2.00 ‰ difference between the two shell δ^18^O datasets (Fig 5), it is necessary to assume implausible δ^18^O_w_ water signals for the two environments. According to Eq 1, the most positive and the most negative δ^18^O values of the inoceramids and the rudists, respectively, would yield the same paleowater temperature if the surface δ^18^O water equaled -3.00 ‰ (or, otherwise, a benthic δ^18^O_w_ = 1.00 ‰). According to the Late Cretaceous freshwater mixing line developed the western Interior Seaway waters [100], such a scenario implies an extraordinary input of freshwater/precipitation to the shallow environment. Accordingly, surface salinity should have been as low as 26 PSU, whereas, normal marine salinity conditions (34 PSU) would have existed at the bottom of the sea. Surface hypohyaline conditions, however, contrast with the highly diverse fully marine Gosau fauna at the studied locality [63,64]. Furthermore, such strong vertical gradient in salinity may have resulted in a density-driven stratification of the water column and reduced levels of oxygen in the benthic environment, as in the case of Western Interior Seaway in North America [100]. Stagnation and benthic hypoxia, however, are not consistent with the diverse macrofossil community of the ‘lastame’ lithofacies [66–72] and the presence of authigenic hematite, which indicate well-oxygenated conditions at the seafloor [18]. Therefore, it is reasonable to assume that the observed δ^18^O difference between the studied rudists and inoceramids resulted from a combined effect of changing water temperature and δ^18^O_w_ signature.

Assuming modern-like vertical δ^18^O_w_ changes for the upper 900 m of the studied region, the proposed model yielded temperature gradients ranging between ca. 14 and 19 °C. Among the modeled scenarios, the most conservative ΔT estimates (13–14 °C; Late Cretaceous and Indian Ocean settings) were very close to the ΔT value (14–15 °C) computed with a homogeneous δ^18^O_w_ value. Therefore, we interpret the vertical effect of changing δ^18^O_w_ on the measured δ^18^O_shell_ amplitude as minor, and ascribe the isotopic differences between the rudist and inoceramid data to a heterogeneous thermal structure of the water body (Fig 5). The precise estimation of the vertical ΔT gradient, however, also depends on the assumed water depth for the Trento Plateau. The oxygen-rich water conditions postulated for the ‘lastame’ lithofacies [67] suggest that the studied environment was located above the oxygen minimum zone [72], which in tropical oceans can be generally positioned between 150 and 500 m water depth [113]. This range fits well with the shallower paleodepths assumed for the Trento Plateau in previous studies [67,72] and make a bathyal depth postulated by others unrealistic [71]. The presence of a diverse ratiolitid rudist fauna in the Scaglia Rossa deposits of Sant´Anna d´Alfaedo, however, provide a strong line of evidence that the studied nodular ‘lastame’ lithofacies was placed in the upper part (~150 m or slightly below) of this bathymetric range [72]. At a water depth between 150 and 200 m, the model suggested ΔT gradients between 13–14 (Late Cretaceous and Indian Ocean), 14–15 °C (Atlantic Ocean and Gulf of Mexico) and 16–17 °C (Pacific Ocean), which compare well to the vertical ΔT values observed in the modern tropical latitudes 10°N (Fig 6C). Previous studies have postulated a poleward shift of ca. 5°–10° for the tropic/sub-tropic belts during the Late Cretaceous [114]. The annual ΔT amplitudes inferred for the shallow water setting (ca. 30 °N) compare well with those observed in modern tropical waters (20°-25 °N) and seem to fit in this frame. However, even in the context of the Late Cretaceous greenhouse world, it seems very unlikely that the near-equatorial scenario could have extended up to studied latitudes. Therefore, to explain the δ^18^O difference between the rudists and inoceramid shells, we hypothesize the existence a heterogeneous structure of the water column at the northwestern Tethyan margin with a bottom source of cold hydrological masses. To enable an easy comparison with the literature data, in the following, temperature estimates will be reported assuming a seasonally constant δ^18^O_w_ value of -1.0 ‰.

The temperature estimates obtained from rudist shells (24–35 °C) are in the range of those reconstructed from fish tooth apatite of the region [21] (Fig 5). This suggests that surface conditions and atmospheric variability affected both the shallow water biotope and the upper layer of the Tethys Ocean (Fig 5). Such warm surface Tethyan-like conditions also likely pervaded the shallow water and shelf environments at higher latitudes at least during part of the Turonian, as suggested by the δ^18^O data of brachiopods [12], rudist [84] and inoceramid shells [55] from Central European Basins. Short intervals of warm water conditions in the European mid-latitudes are also supported by the northward extension of the biogeographic province of Tethyan ammonites [13]. On the contrary, the benthic water temperatures of the Trento Plateau are close to the conditions reconstructed for the shelf environments of Central Europe during the Hitch Wood Event (Fig 5). The Hitch Wood Event repents a short time interval of the Late Turonian during which boreal water masses spread into Central European basins and caused a continental-wide cooling of shelf sea waters [13,115]. Furthermore, an influence of water masses with boreal affinity on the Trento Plateau during the late Turonian–early Coniacian is compatible with the more or less coeval begin of deep water production in the North Atlantic [9,10], the inflow of oxygen-rich waters in the western sub-basins of the Tethys [115,116] and the increased uptake of unradiogenic neodymium isotopes in apatite of fish teeth of the Tethys [14].

The described thermal decoupling between bottom and shallow water habitats contrasts with the existence of an active upwelling system at the northwestern margin of the Tethys postulated by previous authors [14]. Although the published neodymium isotope data suggest some degree of vertical exchange in water column, our seasonally resolved temperature estimates clearly indicate that bottom waters did not reach the shallow water environment of the Helvetic Shelf. Furthermore, existing studies on shallow water filter-feeding bivalves show that exposure to upwelling events usually results in a linear relationship between δ^13^C and δ^18^O values of the shells [117–119]. Such a relationship, however, was not detected in the stable isotope values of the *V*. *inaequicostatus* (Figs 3A and 4A), for which a heterotrophic lifestyle can be assumed. The lack of a correlation between shell δ^13^C and δ^18^O values is typical for filter-feeding bivalves from shallow water habitats, where nutrient supply, primary production and water temperatures are controlled by surface seasonality [102]. The shell growth rates of the photosymbiotic *H*. *resectus* provide a further line of evidence for the shallow water environment being mainly controlled by surface dynamics. The metabolic response to heat stress among bivalves is heterogeneous. However, taxa able to grow their shells over water temperature changes of >12 °C are not uncommon (97,102). Following tank experiments short-term temperature rises by up to 6 °C can induce death of the symbiotic zooxanthellae and bleaching [120,121], without presenting a major metabolic stress for the bivalve host [120]. Furthermore, as discussed above, the annual ΔT amplitude of the Tethyan shallow water might have been lower than 11 °C, considering the effect of the seasonally changing δ^18^O_w_ on the δ^18^O_shell_ records of the rudists. Hence, it is likely that fluctuations in water temperatures did not represent a major obstacle for *H*. *resectus* to biomineralize. On the contrary, nutrient availability and irradiance are the two major limiting factors for shell growth in algae-bearing bivalves [45]. Since accretion rates in *H*. *resectus* decreased during summer (i.e., maximum seasonal irradiance), it is plausible that growth was mainly controlled by nutrient limitation. In this context, increased summer insolation might have boosted environmental photosynthetic activity leading to nutrient depletion of the upper photic zone in a comparable manner as observed in modern sub-tropical zones [122].

The positive correlation between δ^13^C and δ^18^O values of the inoceramids suggests that the process governing temperature fluctuations also affected food availability and/or the stable isotopic signature of the DIC on the Trento Plateau. The concomitant increase in food availability and temperature on the seafloor resulting from snowing of organic matter and advection of water masses from the surface has been invoked to explain the observed stable isotope pattern in previous Late Cretaceous studies [43,53]. However, as observed on modern basinal highs [123], seasonal changes in horizontal transport of particulate organic matter might have also played a role in the trophic balance of the environment. Additionally, bottom currents from the North Atlantic possibly provided a steady supply of cold and nutrient-rich waters to the lower photic zone, where weakened insolation determined coincidental fluctuations in seasonal primary production and water temperatures. If this interpretation holds true, the present sclerochronogical data possibly suggest, to some extent, a decoupling also in the food webs between the shallow and bottom environments at the northwestern margin of the Tethys.

## Conclusions

The present study highlights the potential of bivalve sclerochronology for Late Cretaceous paleoclimate reconstructions. Whereas most of the existing sclerochronological studies have focused on shallow water bivalves, information on the seasonality in deeper settings during this time interval is still very limited. As shown here, seasonally resolved environmental data from different taxa, which inhabited different water depths, can provide quantitative information on the internal structure of an ancient water column. Such data are often only available from numerical climate simulations and are of fundamental importance for paleoceanographic studies.

Our findings indicate the existence of a steep vertical temperature gradient of ca. 15 °C from the bottom to the surface of the northwestern Tethys Ocean during the late Turonian-early Coniacian. Such gradient is steeper than observed at modern tropical latitudes (20°N–25°N) under normal marine conditions and suggest the existence of a structurally heterogenous water body at the northwestern Tethyan margin. Bottom cooling was likely caused by benthic currents with old and nutrient-rich waters of North Atlantic origin. At the same time, however, strong radiative forcing at the surface resulted in the warmest conditions recorded over the entire Late Cretaceous in the shallow water habitats of the region. The substantial differences in the vertical water temperatures and their seasonal amplitudes suggest the existence of a perennial thermocline. The precise position of the latter remains unknown, however, it was likely located below the migratory depth of the ichthyofauna used for δ^18^O-based temperature reconstructions [21]. Moreover, the stable isotopic patterns of the studied bivalve shells also suggest that the supply of North Atlantic waters to the lower photic zone may have resulted in a certain degree of independence between the trophic systems of the two environments. Hence, it is unlikely that an active upwelling system persisted in the region at that time, as postulated by previous authors.

## Supporting information

S1 Fig (JPG)

S1 Data (XLSX)
