# Supplementary figures and images for: Paleoceanography of the Late Cretaceous northwestern Tethys Ocean: Seasonal upwelling or steady thermocline?
Source: PLoS One. 2020 Aug 27;15(8):e0238040. doi: 10.1371/journal.pone.0238040 (PMC7451568; doi:10.1371/journal.pone.0238040)

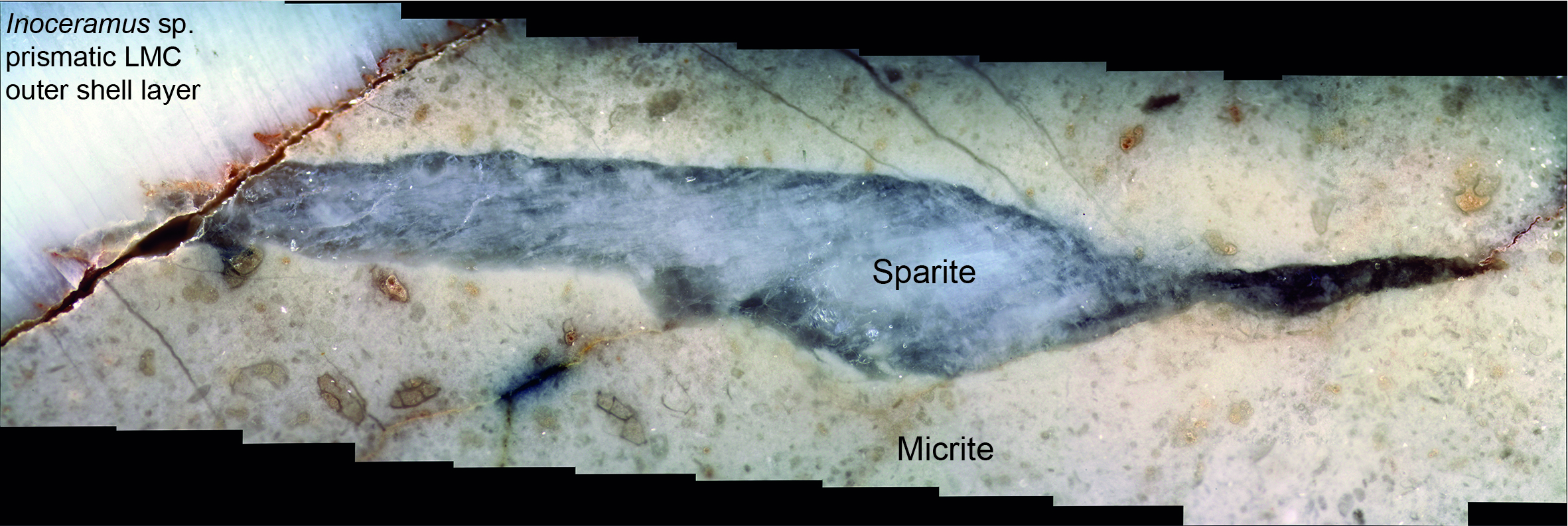

Supplement: S1 Fig — (JPG) [file pone.0238040.s001.jpg]
